# Supplementary material for: Organ pedalboard as a rehabilitation tool: A qualitative exploratory study of healthcare providers’ perceptions and recommendations
Source: PLoS One. 2024 Dec 19;19(12):e0314147. doi: 10.1371/journal.pone.0314147 (PMC11658632; doi:10.1371/journal.pone.0314147)
Supplement: S1 File — (DOCX) [file pone.0314147.s001.docx]

**S1 File. COREQ (COnsolidated criteria for REporting Qualitative research) Checklist**

| **Topic** | | **Item No.** | **Guide Questions/Description** | **Reported on Page No.** |
| --- | --- | --- | --- | --- |
| **Domain 1: Research team and reflexivity** | |  |  |  |
| *Personal characteristics* | |  |  |  |
| Interviewer/facilitator | | 1 | Which author/s conducted the interview or focus group? | 6 |
| Credentials | | 2 | What were the researcher’s credentials? E.g. PhD, MD | 6, 9 |
| Occupation | | 3 | What was their occupation at the time of the study? | 6, 9 |
| Gender | | 4 | Was the researcher male or female? | 6, 9 |
| Experience and training | | 5 | What experience or training did the researcher have? | 6, 9 |
| *Relationship with participants* | |  |  |  |
| Relationship established | | 6 | Was a relationship established prior to study commencement? | 6 |
| Participant knowledge of the interviewer | | 7 | What did the participants know about the researcher? e.g. personal goals, reasons for doing the research | 6 |
| Interviewer characteristics | | 8 | What characteristics were reported about the inter viewer/facilitator? e.g. Bias, assumptions, reasons and interests in the research topic | 6 |
| **Domain 2: Study design** | |  |  |  |
| *Theoretical framework* | |  |  |  |
| Methodological orientation and Theory | | 9 | What methodological orientation was stated to underpin the study? e.g. grounded theory, discourse analysis, ethnography, phenomenology, content analysis | 8 |
| *Participant selection* | |  |  |  |
| Sampling | | 10 | How were participants selected? e.g. purposive, convenience, consecutive, snowball | 6 |
| Method of approach | | 11 | How were participants approached? e.g. face-to-face, telephone, mail, email | 6 |
| Sample size | | 12 | How many participants were in the study? | 9 |
| Non-participation | | 13 | How many people refused to participate or dropped out? Reasons? | 9 |
| *Setting* | |  |  |  |
| Setting of data collection | | 14 | Where was the data collected? e.g. home, clinic, workplace | 6 |
| Presence of non participants | | 15 | Was anyone else present besides the participants and researchers? | 6 |
| Description of sample | | 16 | What are the important characteristics of the sample? e.g. demographic data, date | 6, 9, Table 1 |
| *Data collection* | |  |  |  |
| Interview guide | | 17 | Were questions, prompts, guides provided by the authors? Was it pilot tested? | 6-7 |
| Repeat interviews | | 18 | Were repeat interviews carried out? If yes, how many? | 7 |
| Audio/visual recording | | 19 | Did the research use audio or visual recording to collect the data? | 7 |
| Field notes | | 20 | Were field notes made during and/or after the interview or focus group? | 7 |
| Duration | | 21 | What was the duration of the inter views or focus group? | 6 |
| Data saturation | | 22 | Was data saturation discussed? | 6 |
| Transcripts returned | | 23 | Were transcripts returned to participants for comment and/or correction? | 9 |
| **Domain 3: analysis and findings** |  | |  |  |
| *Data analysis* |  | |  |  |
| Number of data coders | 24 | | How many data coders coded the data? | 9 |
| Description of the coding tree | 25 | | Did authors provide a description of the coding tree? | 9 |
| Derivation of themes | 26 | | Were themes identified in advance or derived from the data? | 9 |
| Software | 27 | | What software, if applicable, was used to manage the data? | 9 |
| Participant checking | 28 | | Did participants provide feedback on the findings? | 9 |
| *Reporting* |  | |  |  |
| Quotations presented | 29 | | Were participant quotations presented to illustrate the themes/findings? Was each quotation identified? e.g. participant number | 11-23 |
| Data and findings consistent | 30 | | Was there consistency between the data presented and the findings? | 11-23 |
| Clarity of major themes | 31 | | Were major themes clearly presented in the findings? | 11-23 |
| Clarity of minor themes | 32 | | Is there a description of diverse cases or discussion of minor themes? | N/A |

Developed from: Tong A, Sainsbury P, Craig J. Consolidated criteria for reporting qualitative research (COREQ): a 32-item checklist for interviews and focus groups. *International Journal for Quality in Health Care*. 2007. Volume 19, Number 6: pp. 349 – 357
