# Supplementary material for: Organ pedalboard as a rehabilitation tool: A qualitative exploratory study of healthcare providers’ perceptions and recommendations
Source: PLoS One. 2024 Dec 19;19(12):e0314147. doi: 10.1371/journal.pone.0314147 (PMC11658632; doi:10.1371/journal.pone.0314147)
Supplement: S2 File — (DOCX) [file pone.0314147.s002.docx]

**S2 File- Interview Guide**

**Awareness**

1. Tell me your understanding of music's role in rehabilitation.
2. Tell me your understanding of using organ/organ pedalboard for rehabilitation.
3. Generally, do you think pedal training can be used as a rehabilitation tool for patients with lower extremity problems? How? Why? Can you elaborate more?

**Perceived enablers**

1. Do you think pedal training can influence people's health? How?
2. (Prompt: what about foot range of motion? what about the foot muscle strength? what about the lower extremity’s muscle power? Anything else?

Prompt: what about physical health? what about functions of the foot? what about motor functions? what about cognitive functions? what about psychological health? what about quality of life?)

1. Which type of patients do you think can benefit from pedal training? How? Any other benefits?

**Perceived barriers**

1. In your opinion, which type of patients may not benefit from the pedal training? Why?
2. Do you think pedal training can be contraindicated for some conditions? Which/why?
3. What are the contraindications for using pedal training in rehabilitation for patients with lower extremity problems? Why?
4. Which type of patients do you think will be reluctant to get pedal training? Why?
5. Which type of patients do you think will be difficult for them to practice pedal training as a rehabilitation intervention? Why?
6. What do you think about the cost of the pedalboard (around HKD$7000) if your organization considers using it for patients with lower extremity problems?
7. What do you think about the size of the pedalboard for a regular clinic in Hong Kong? Any considerations for the space? Any other factors? Any suggestion about the pedalboard design?

**Attitudes**

1. What do you suggest to adapt the demonstrated pedal training to make it suitable and applicable for rehabilitating patients with lower extremity problems? Why?

*If the named barriers can be eliminated and modifications are made…*

1. As a healthcare provider, how likely you will use pedal training in rehabilitation for patients with lower extremity problems? *(0= very unlikely; 10= very likely)*
2. Which discipline of the healthcare providers will be most likely to use pedal training in their patients? Why?
3. If pedalboard is available in your clinical setting, which type of patients do you intend to test the pedal training on? Which age group of people you will likely to use pedal training in?
4. If the pedal training proved to be effective for certain lower extremity conditions, will you recommend to your colleagues to use pedal training for similar lower extremity problems? Why?
5. What will make you use the pedal training for managing lower extremity problems?
6. What will make you recommend the pedal training for your peers for managing lower extremity problems?
