# Supplementary material for: Organ pedalboard as a rehabilitation tool: A qualitative exploratory study of healthcare providers’ perceptions and recommendations
Source: PLoS One. 2024 Dec 19;19(12):e0314147. doi: 10.1371/journal.pone.0314147 (PMC11658632; doi:10.1371/journal.pone.0314147)
Supplement: Attachment — Submitted filename: Response to Reviewers.docx [file pone.0314147.s003.docx]

PONE-D-24-14802

Organ pedalboard as a rehabilitation tool: a qualitative exploratory study of healthcare providers’ perceptions and recommendations

16 September 2024

Dear editor and reviewers,

We would like to thank you for taking the time to review our manuscript and provide constructive comments to facilitate the revision of our manuscript. We addressed the comments point-by-point in the below and uploaded a revised manuscript with and without tracked changes. The major change we made was to include a Biopsychosocial Model to guide our study.

Regarding data availability, we also wish to confirm that “All relevant data are within the manuscript and its Supporting Information files.” Our submission does not contain all raw data and we do not upload them in any public repository. The qualitative nature of our study preserve little meaning for replication and that relevant data are already reported in our study.

We hope the revised version of our manuscript meet the standard for publication and look forward to hearing from you.

Kind regards,

Dr. Fadi Al Zoubi

Assistant Professor

The Hong Kong Polytechnic University

**Responses to associate reviewers' comments:**

| **Comments** | **Responses** |
| --- | --- |
| Reviewer #1: I would like to thank the authors for reporting such an interesting and novel study. I read through the manuscript with great interest, and I think it has the potential to extend our knowledge about music-based rehabilitation with organ pedalboard. However, due to some issues listed below, there is still room for improvement in the manuscript. | Thank you for your thoughtful and encouraging feedback. We appreciate your interest in our study and your recognition of its potential to advance knowledge in music-based rehabilitation with the organ pedalboard. We have carefully addressed the issues you have highlighted to improve the manuscript. |
| 1. Background, theoretical framework (if any), and rationale for the study can be strengthened. |  |
| The authors have listed out some previous findings about the treatment effects of music-based intervention for rehabilitation. However, it remains unclear about why the authors would like to target lower extremity conditions, which seemed to be a relatively broad category. It would also be important to put more emphasis on the review of previous findings on existing music-based intervention for lower extremity conditions. This can help to identify the research gap. | Thank you for your suggestion. We include the following text in **Introduction (page 4, lines 69-80):**  “The therapeutic effects of music-based interventions have been extensively studied, particularly in relation to neurorehabilitation, where such interventions have demonstrated efficacy in improving motor and cognitive functions [9, 10]. Our focus on lower extremity conditions arises from the observation that rehabilitation of these conditions, such as stroke-related gait impairments, often necessitates interventions that are both motivating and conducive to sustained engagement. While lower extremity conditions encompass a wide range of impairments, including those affecting balance, gait, and overall mobility [11], this diversity underscores the need for innovative and versatile rehabilitation tools capable of addressing various challenges within this category. Music-based interventions, particularly those that incorporate rhythm and movement coordination, have shown promise in enhancing rehabilitation outcomes for these impairments, making them a compelling area for further research [12-14].” |
| Similarly, it would be essential to state why organ pedalboard was chosen as a potential rehabilitation tool. What makes it unique or more suitable than other musical instruments? | Thank you for your suggestion. We include the following text in the **Introduction (page 7, lines 131-139):**  “The selection of the organ pedalboard as a rehabilitation tool is grounded in its unique ability to engage the lower extremities in a musically and rhythmically structured environment. Unlike many other musical instruments, the organ pedalboard requires precise, coordinated foot movements to produce sound, thereby directly targeting motor control and coordination of the lower limbs [23]. This makes it a particularly well-suited for rehabilitation applications. The repetitive goal-oriented nature to improve functional outcomes through tasks that mimic daily activities.” |
| In addition, the rationale for selecting physiotherapists, occupational therapists, music therapists, and sports trainers as participants should be added. It would also be helpful to provide background information about the rehabilitation work of these four groups of health professionals in clinical and community settings so that readers may differentiate them from each other. | We include a paragraph to elaborate on the rationale of multidisciplinary approach and the role of each discipline in **Participants and sampling** under **Methods (pages 9-10, lines 201-211):**  “The rationale for selecting physiotherapists, occupational therapists, music therapists, and sports trainers as participants in this study is well-justified, as these healthcare professionals play complementary roles in the rehabilitation process, each bringing unique expertise that can provide valuable insights into the potential applications of the organ pedalboard intervention. Physiotherapists specialize in restoring physical function and mobility, occupational therapists focus on enabling individuals to participate in meaningful daily activities, music therapists utilize therapeutic potential of music to address a wide range of needs, and sports trainers optimize physical performance and prevent sports-related injuries. This multidisciplinary approach ensures that the intervention is assessed from diverse professional perspectives, thereby enhancing the validity and applicability of the findings in both clinical and community settings.” |
| 2. Issue about research objectives. |  |
| The authors may note that the objectives stated in Abstract (Lines 25-28) are not fully consistent with those in Introduction (Lines 90-95). Also, the section of Discussion should start with a paragraph to summarize major findings in response to each research objective. | **Revised objectives in the Abstract (page 2, lines 25-29):**  “This qualitative explored healthcare providers' perceptions and attitudes regarding the use of organ pedalboards as a rehabilitation tool, particularly for lower extremity conditions. The study also sought to identify the perceived barriers and facilitators in adopting organ pedalboards within rehabilitation settings, as well as to gather healthcare providers' recommendations for integrating this tool into clinical practice.”  **Added paragraph in the Discussion (page 31, lines 670-681):**  “We addressed the research objectives outlined in the introduction and successfully identified healthcare providers' perceptions of using organ pedalboards as a rehabilitation tool for lower extremity conditions. The findings revealed that healthcare providers view music as a motivator that enhances patient engagement and supports natural movement, particularly in lower limb rehabilitation. Additionally, the study highlighted key barriers and facilitators influencing the adoption of organ pedalboards in clinical practice, such as safety concerns and the need for customized training programs. Importantly, the healthcare providers provided specific recommendations on how organ pedalboard training could be integrated into existing rehabilitation protocols, emphasizing its potential to improve physical, cognitive, and psychosocial outcomes. These insights contribute significantly to the development of targeted rehabilitation strategies and underscore the need for further research to optimize the use of organ pedalboards in clinical settings.” |
| 3. Method |  |
| The rationale for adopting focus group interviews should be provided. The author may also explain why each focus group included different healthcare providers, instead of having focus groups with providers from the same discipline. What group dynamics were expected and observed? | We elaborate this comment by adding the following text under **Design (page 8, lines 162-175):**  “This qualitative study employed focus group interviews to explore healthcare providers' perceptions of using organ pedalboards as a rehabilitation tool for lower extremity conditions. The decision to use focus group interviews was intentional, as this method allows participants to interact, build on each other 's ideas, and generate deeper insights through group dynamics. Furthermore, the focus groups were designed to include healthcare providers from different disciplines, such as physiotherapists, occupational therapists, music therapists, and sports trainers. This interdisciplinary composition was a deliberate choice, as it was expected to foster diverse perspectives and encourage cross-disciplinary dialogue, yielding a richer understanding of the potential benefits and challenges of integrating organ pedalboard into rehabilitation practice. By bringing together professionals with varied expertise and backgrounds, the focus group discussions were anticipated to uncover nuanced insights that may not have emerged from individual interviews or more homogeneous group settings, stimulating new ideas, challenging assumptions, and providing a comprehensive exploration of the topic from multiple angles.” |
| During each focus group interview, it seemed that a considerable proportion of time was spent on demonstration and trials of organ pedalboard. How long was the discussion among participants? | The discussion lasted for about 50-60 minutes. We clarify this in **Data collection (page 10, lines 219-227):**  “Each focus group session lasted approximately 1 hour and 15 minutes and followed a structured flow. The session began with a 5-minute demonstration of the organ pedalboard, followed by a 10-minute period where participants had the opportunity to personally try out the device. The majority of the session, spanning 50-60 minutes, was then dedicated to a semi-structured discussion guided by open-ended questions. This discussion-focused portion allowed the researchers to thoroughly explore the participants' perceptions, attitudes, and perceived barriers to using the organ pedalboard in rehabilitation settings. The semi-structured approach provided flexibility to probe deeper into relevant topics as they arose.” |
| As some participants knew the lead researcher personally, the authors may disclose more details about personal reflexivity regarding this point. | To address potential bias due to pre-existing relationships between some participants and the lead researcher, MK, the study employed several measures. We explain such in the second paragraph of **Reflexivity (pages 14-15, lines 308-318):**  “Reflexivity was integral to the ethical conduct of this research, as it helped minimize power dynamics and ensure the participants' voices were accurately represented, leading to balanced and objective conclusions. Given that some participants were personally acquainted with the lead researcher (MK), particular measures were employed to minimize potential bias. All participants were explicitly informed of the study's objectives and the distinct role of MK as a facilitator, separate from any personal connection. Participants were encouraged to provide honest and candid feedback, independent of their relationship with MK. Additionally, transparency was reinforced by emphasizing the structured nature of the interview process, which strictly adhered to the interview guide developed by the research team. During the interviews, MK maintained a neutral stance and carefully managed her influence to minimize bias.” |
| Since “lower extremity conditions” is a broad concept, it could be difficult to reach data saturation. It would be helpful if the authors can report how data saturation was achieved. | We address this in **Participants and sampling (page 9, lines 183-186):**  “To address the broad concept of "lower extremity conditions" and achieve data saturation, we continued sampling until no new themes emerged from the interviews. This was monitored closely through ongoing data analysis and was confirmed when subsequent interviews added no new significant insights.” |
| The study appeared to be a semi-structured focus group interview, rather than a structured interview. | Thank you. The terms are updated. See page 2, line 35; page 10, lines 223 and 226; page 11, line 229. |
| 4. Findings |  |
| Following COREQ, please name the section as “Findings” instead of “Results”. | Thank you. The terms are updated. See page 2, line 40 and page 15, line 320. |
| The major themes emerged from the focus group interviews are nicely presented. It would be better to identify sub-themes so that readers can capture the findings more easily. | Thank you for your suggestion. After consideration, we think that the sub-themes should remain unchanged but we decide to add three higher-order themes to categorize sub-themes which may make readers easier to understand our findings. We add the following text **(pages 17-18, lines 343-368)**:  “The analysis of the focus group data revealed three higher-order themes that provide a structured framework to better categorize and clarify the sub-themes that emerged. These higher-order themes enhance the readability and understanding of the research findings.  The first higher-order theme is "**The Multifaceted Role of Music in Rehabilitation**", which encapsulates the overarching influence of music within the rehabilitation context. It includes sub-themes that explore the role of music as a key motivator, encouraging sustained patient engagement and natural movement. Additionally, this theme examines how the organ pedalboard is perceived not only as a therapeutic device but also as a musical instrument, making the rehabilitation process more engaging and less clinical for patients.  The second higher-order theme is "**Physical, Cognitive, and Psychosocial Benefits of the Organ Pedalboard**", which brings together the various benefits of using the organ pedalboard in rehabilitation. Sub-themes under this category include physical benefits, such as improvements in range of motion, flexibility, balance, and coordination; cognitive benefits, including enhancements in memory, attention, and cognitive processing; as well as the psychosocial benefits, where the pedalboard boosts patient confidence, provides a sense of accomplishment, and facilitates social interaction.  The third higher-order theme is "**Practical Considerations and Safety in Implementing Pedalboard Training**"**,** which addresses the logistical and safety aspects of incorporating the organ pedalboard into rehabilitation programs. This theme includes sub-themes that discuss the unique requirements and adaptations necessary for effective use of the pedalboard across different patient populations, the importance of safety features and modifications to prevent injury, and the need for programming and customization to make the pedalboard a more accessible and effective tool in rehabilitation settings.” |
| Please consider reporting participants job status (full-time or part-time) and the population of their patients in Table 1. | We add the information of job status and population of patients as suggested in Table 1 and in the text accordingly **(pages 15-16, lines 343-338)**:  “The majority of the participants (70%, n = 12) were employed full-time across a variety of healthcare settings, including hospitals (n = 6), elderly care homes (n = 6), private clinics (n = 2), and other facilities (n = 3). The participants primarily served older adult (n = 10), adult (n = 5), and paediatric (n = 2) patient populations.” |
| 5. Discussion |  |
| According to the interview guide, the high cost of organ pedalboard was listed as a potential barrier. It seemed counter-intuitive that this was not included in Findings. Moreover, the modification and programming of organ pedalboard to address the barriers would result in even higher cost. The authors may discuss this point. | Following your suggestion, we insert a new paragraph in **Discussion (pages 35-36, lines 773-784)**:  “The cost of implementing organ pedalboards in rehabilitation is indeed a critical factor that must be acknowledged. While our study focused on the potential benefits of this tool, the oversight of not emphasizing the high cost, as highlighted in the interview guide, should be addressed. The participants' primary focus was on the therapeutic advantages rather than the financial barriers. However, the modification and programming costs associated with adapting the pedalboard for rehabilitation could pose significant challenges, particularly in resource-limited settings. To mitigate these concerns, it is important to clarify that the pedalboard is not intended for individual patient ownership, but rather should be purchased and maintained by institutions or clinical settings, such as hospitals and research institutes, where patients would undergo training. This institutional ownership approach can help ensure broader accessibility and address the individual financial burdens, allowing the focus to remain on the clinical benefits of this rehabilitation tool.” |
| Also, since organ pedalboard is not a commonly used musical instrument, could there be cultural barriers in adopting it for rehabilitation? Would patients prefer musical instruments that they are familiar with? | The organ's unfamiliarity as an instrument in Hong Kong may pose cultural barriers to adoption, but the novelty could also pique patient interest and facilitate engagement. The current lack of familiarity may stem from limited exposure rather than inherent aversion. Importantly, future research should explore patients' perceptions and experiences with the organ pedalboard directly to understand their perspectives and inform effective integration strategies. We add the following paragraph **(page 36, lines 786-805)**:  “The cultural context must be carefully considered when evaluating the adoption of the organ pedalboard as a rehabilitation tool. In the Hong Kong settings where the study was conducted, the organ pedalboard is not a commonly used musical instrument. This lack of familiarity may create cultural barriers, as patients may prefer to work with instruments they are more accustomed to, such as the piano or traditional Chinese instruments. These cultural preferences could impact the effectiveness of the intervention, as patient engagement is closely linked to their comfort and familiarity with the therapeutic tools used. However, it is also important not to make assumptions that patients would be unwilling to try the pedalboard. In fact, the novelty of this “new instrument” may actually create a sense of intrigue and interest, potentially facilitating greater patient engagement. The current unpopularity of the pedalboard might be due to factors such as its availability, personal interest, or lack of exposure and training, rather than inherent cultural aversions. To address this challenge, we believe that with institutional ownership and the implementation of "train-the-trainer" programs for healthcare professionals, the organ pedalboard could be effectively integrated into rehabilitation settings. By offering the pedalboard as an option, patients can explore this unique tool as part of their rehabilitation, potentially enhancing their overall experience and outcomes. Importantly, future research should also explore patients' perceptions, attitudes, and experiences with the organ pedalboard directly. Understanding the target population's perspectives, preferences, and cultural considerations will be crucial in developing effective strategies for the successful integration of this rehabilitation tool.” |
| The authors may also try to interpret the findings in the sociocultural context of Hong Kong and see if they are culturally specific or generalizable to other communities. | Thank you for your suggestion. We add the following text **(page 37, lines 807-815)**:  “The findings offer valuable insights, but they should be interpreted within Hong Kong's sociocultural. Generalizability to communities with different cultural backgrounds or healthcare systems may be limited, as Hong Kong is a developed region where Western therapies are widely accepted. We believe that there are minimal barriers to pedalboard rehabilitation adoption in similar developed regions globally. However, future research should explore the applicability in diverse cultural settings to better understand the broader potential and limitations of this approach. Such cross-cultural investigations will provide crucial insights into how the organ pedalboard intervention may need to be tailored or adapted to ensure its effectiveness and acceptability in varied settings. ” |

**Reviewer #2: August 7, 2024**

| **Comments** | **Responses** |
| --- | --- |
| Comment on the Introduction |  |
| Dear Authors,  Thank you for the opportunity of reading your manuscript titled “Organ pedalboard as a rehabilitation tool: a qualitative exploratory study of healthcare.” The primary aim of the study is to explore the feasibility of using organ pedal training as a rehabilitation tool for patients with lower extremity problems. The researchers sought to understand healthcare providers' perceptions regarding the use of organ pedalboards in rehabilitation, including the potential benefits, attitudes, and perceived barriers associated with this innovative approach. The study's findings reveal that healthcare providers view organ pedalboards as a promising rehabilitation tool, particularly for lower extremity rehabilitation. They perceive music as a significant motivator that facilitates natural movement through rhythm and beats, enhancing patient engagement and psychosocial well-being. The pedalboard training is reported to improve range of motion, balance, and coordination, while also offering cognitive benefits. However, safety concerns regarding its implementation were acknowledged, highlighting the need for careful planning. Overall, the insights from healthcare providers can inform the development of a structured training protocol that accommodates various physiological movements, thereby enhancing rehabilitation practices. The study is notable for its innovative approach in exploring the use of organ pedalboards for rehabilitation. It comprehensively examines physical, cognitive, and psychosocial benefits, involving diverse healthcare providers to gather a wide range of insights. The qualitative methodology allows for in-depth understanding, while practical recommendations address safety and usability concerns. By identifying barriers and facilitators, the study provides valuable guidance for clinical implementation and sets a foundation for future research, potentially benefiting a broad range of patients.  The paper would benefit from the inclusion of a conceptual framework, a section on reflexivity, and pieces of evidence to enhance the credibility of the results. This can be achieved through member checks or peer debriefing. Please review the comments provided below. | Thank you for the comprehensive feedback on our manuscript. We appreciate the reviewer's constructive comments and addressed them as follows below. |
| 1. Could you provide information about the study design and numerical results? It will give the readers idea on the quality of evidence being presented. At the point in the text that reads “another study reported enhanced muscle endurance in middle-aged adults performing lower limb exercises while listening to music [15]. These findings underscore the potential of music intervention to divert attention from discomfort and reduce perceived exertion levels [16-18], highlighting its prospective utility in rehabilitation settings.” | Thank you for your comment. We refine this paragraph as below **(page 5, lines 85-105)**:  “Another study [16] investigated the effects of self-selected versus motivational music on muscle endurance and affective state in middle-aged adults. The study employed a within-subject design where 26 healthy middle-aged males (average age 50.8±8.4 years) performed maximal and endurance isometric strength tests under three different conditions: self-selected music (SSM), motivational music (MM), and a control condition (CC) with no music. The results showed that mean force during the isometric endurance test was significantly higher in the SSM condition (507.3±132.2 N) compared to both MM (476.3±122.4 N, p<0.01) and CC (484.6±119.2 N, p=0.03). Additionally, participants reported a higher Felt Arousal Scale (FAS) score and Feeling Scale (FS) score in the SSM condition, indicating a more positive affective state. These findings suggest that self-selected music can enhance muscle endurance and positively influence mood during strength exercises in middle-aged adults. These findings underscore the potential of music interventions to divert attention from discomfort and reduce perceived exertion levels. For instance, Magill-Levreault [17] highlighted the role of music therapy in pain management, where it was shown to decrease pain perception by altering affective and cognitive processes. Similarly, Mohammadzadeh et al. [18] found that music significantly reduced the rate of perceived exertion (RPE) during progressive exercise, especially among untrained individuals, while Potteiger et al. [19]demonstrated that various types of music, including self-selected music, were associated with lower RPE during moderate-intensity exercise. These studies collectively highlight the prospective utility of music in rehabilitation settings, where managing discomfort and perceived exertion is crucial.” |
| 2. Could you please provide evidence, whether direct or indirect, as reported in the literature, on the effectiveness of organ pedaling in treating lower extremity conditions? | Literature indirectly support the effectiveness of organ pedaling in treating lower limb conditions. We insert the following text to elucidate **(page 6, lines 109-129)**:  “Evidence supporting the use of organ pedaling as an effective intervention for lower extremity rehabilitation comes from various domains where music and movement are integrated into therapeutic practices. Organ pedal training, which involves coordinated lower limb movements similar to those required in other rhythmic and music-based therapies, has shown promise in enhancing motor functions and overall rehabilitation outcomes. For instance, music therapy has been widely recognized for its positive impact on motor recovery in neurological conditions such as stroke and Parkinson’s disease, where rhythmic auditory stimulation significantly improves gait and mobility [20, 21]. This suggests that the rhythmic and repetitive nature of organ pedaling could similarly benefit lower extremity rehabilitation. Moreover, the physical demands placed on organists, who frequently engage in complex foot movements, are comparable to those seen in athletic training. This has led to observations that organists develop enhanced lower limb strength, endurance, and coordination, further supporting the potential of organ pedalboard as a rehabilitation tool [22]. Additionally, music’s ability to serve as a motivator during physical activity, reducing perceived exertion and increasing endurance, has been well-documented in exercise settings [18, 19]. These findings are indicative of the broader applicability of music-integrated physical exercises in promoting lower limb health. Although direct studies on organ pedaling are limited, the extrapolation from related music and movement therapies strongly supports its potential effectiveness in treating lower extremity conditions. The unique combination of rhythmic movement and cognitive engagement in organ pedaling makes it a promising area for further research and application in rehabilitation settings.” |
| 3. Please provide a conceptual framework or theoretical model (e.g., Normalization Process Theory, Theoretical Framework of User Experience, Biopsychosocial model) to propose the beneficial effects of organ pedaling on lower extremity conditions. Including a conceptual framework in qualitative studies is essential for several reasons. It guides the research process, offering a clear structure for conducting the study and illustrating the relationships between key concepts and variables, thus ensuring focus and coherence. Furthermore, it clarifies the underlying assumptions, theories, and beliefs that shape the research, providing important context and perspective. The conceptual framework influences the choice of research methods and techniques, aligning them with the research objectives and questions. It also strengthens the validity and credibility of the study by grounding it in existing knowledge and theory, making it easier to justify and explain the findings. Additionally, it aids in interpreting the findings by providing a lens through which the data can be understood, linking the results to broader theories and concepts, and making them more meaningful and relevant. Moreover, it communicates the scope and boundaries of the study to the audience, preventing misinterpretations. Finally, it helps identify gaps in existing knowledge and literature that the study aims to address, situating the research within the broader academic discourse and demonstrating its contribution. Overall, a conceptual framework is a foundational element in qualitative research that ensures the study is systematic, coherent, and grounded in existing knowledge and theory. | We decide to include a Biopsychosocial model to guide our study following your suggestion **(pages 7-8, lines 141-158)**:  “To explore the therapeutic potential of organ pedaling for lower extremity rehabilitation, this study retrospectively applied the Biopsychosocial Model [24] as a guiding conceptual framework. The Biopsychosocial Model proposed by Engel (1977), posits that health outcomes result from the intricate interplay between biological, psychological, and social factors. This framework is particularly apt for understanding how organ pedaling might influence lower extremity conditions, as it addresses not only the physical rehabilitation of muscles and joints, but also the psychological and social dimensions of recovery. From a biological perspective, organ pedaling engages multiple lower limb muscle groups, promoting range of motion, flexibility, and strength, which are critical for rehabilitation [20]. Psychologically, the rhythmic and musical nature of pedaling can enhance patient motivation and adherence to rehabilitation protocols by reducing the perception of effort and increasing overall engagement [19]. Socially, the use of music-based interventions such as organ pedaling may facilitate social interaction, improving the overall well-being of patients through shared musical experiences [25]. By incorporating the Biopsychosocial Model, this study aims to provide a comprehensive understanding of the potential benefits of organ pedaling for lower extremity rehabilitation. This framework guides the research process, ensuring that the investigation remains focused on the interconnected biological, psychological, and social factors that contribute to successful rehabilitation outcomes. |
|  |  |
| Comments on the Method |  |
| 1. What are the eligibility criteria regarding experience in utilizing an organ pedalboard? Is it deemed essential? | There was no special requirement regarding participants’ experience on organ pedalboard. We include this sentence in **Participants and sampling (page 9- Lines 189-191)**:  “No prior experience with organ pedalboards was required for participation, as the primary focus was on exploring perceptions and potential applications of organ pedal training rather than assessing technical proficiency.” |
| 2. How was the relationship between these participants and MK, whom they personally know, addressed? At the point in the text that reads “Few initial participants knew MK personally, a female research associate (registered nurse; MA in psychology in music) prior to the research. | To address potential bias due to pre-existing relationships between some participants and the lead researcher, MK, the study employed several measures. We explain such in the second paragraph of **Reflexivity (pages 14-15, lines 308-318)**:  “Reflexivity was integral to the ethical conduct of this research, as it helped minimize power dynamics and ensure the participants' voices were accurately represented, leading to balanced and objective conclusions. Given that some participants were personally acquainted with the lead researcher (MK), particular measures were employed to minimize potential bias. All participants were explicitly informed of the study's objectives and the distinct role of MK as a facilitator, separate from any personal connection. Participants were encouraged to provide honest and candid feedback, independent of their relationship with MK. Additionally, transparency was reinforced by emphasizing the structured nature of the interview process, which strictly adhered to the interview guide developed by the research team. During the interviews, MK maintained a neutral stance and carefully managed her influence to minimize bias.” |
| 3. Have the participants signed an informed consent form? At the point in the text that reads “Participants and sampling.” | Yes, definitely. We add the following text to clarify **(page 9, lines 197-199)**:  “All participants gave written informed consent prior to inclusion. The consent process detailed the study’s aims, procedures, potential risks, and benefits. Participants were also advised of their right to withdraw at any time without repercussions.” |
| 4. A conceptual framework will be useful in understanding the reasons behind the questions during the interview. At the point in the text that reads “Structured interviews were conducted by the lead researcher, MK, who has experience in conducting qualitative research in rehabilitation, using open-ended questions developed by the research team.” | Thank you for your suggestion, we include the following text to elaborate **(page 11- lines 236-241)**:  “The interview questions were guided by the Biopsychosocial Model, which provided a conceptual framework for understanding organ pedal training's potential benefits. This model helped structure the inquiry into how physical, psychological, and social factors might influence the effectiveness of the intervention. The questions were designed to comprehensively explore these dimensions, keeping the discussion focused and relevant to the study's objectives.” |
| 5. This should be included as a study limitation. At the point in the text that reads “The interview guide was not pilot tested.” | We agree with you and include the following text in the second last paragraph of **Discussion (page 37- lines 819-822)**:  “A notable limitation is the interview guide lacked pilot tested before implementation. This could have missed chance to refine questions or structure, potentially affecting data quality and depth. Future studies should pilot the interview guide to identify and address issues prior to full data collection.” |
| 6. Was there any field note-taker during the interview? | The interviews were not accompanied by a dedicated field note-taker. As mentioned in the manuscript, brief notes were made by the researchers conducting the interviews **(lines 234)**. However, these notes were not as detailed or comprehensive as formal field notes that would be generated by a separate note-taker observing the interview process. The lack of dedicated field notes is a limitation of the current study, as it may have resulted in the loss of potentially valuable contextual information and non-verbal observations that could have provided additional insights. We consider this as a study limitation and include the following text in **Discussion (page 37, lines 822-827)**:  “Another limitation of the current study is the absence of comprehensive field notes taken by a dedicated note-taker during the interviews. While the researchers made brief notes throughout the discussions, these did not capture the level of detail and contextual observations that formal field notes would have provided. The lack of a dedicated note-taker may have resulted in the loss of valuable insights into the non-verbal cues, interactions, and nuances of the interview process.” |
| 7. What does C2-G4 mean? | We explain the meaning of C2-G4 by adding the following text in **Organ pedalboard description (page 11, lines 245-249)**:  “The organ pedalboard used in this study spanned a range from C2 to G4, encompassing 32 musical notes on a standard organ. The lowest note was C2, while the highest reached G4, covering approximately two and a half octaves. This extensive range enabled a variety of foot movements and exercises targeting diverse muscle groups in the lower extremities.” |
| 8. What does MIDI mean? | We further explain “MIDI” in **Organ pedalboard description** with the following text **(pages 11-12, lines 251-256)**:  “The pedalboard was equipped with a MIDI (Musical Instrument Digital Interface) function, a standard technology that allows electronic musical instruments, computers, and other equipment to communicate and synchronize with each other. In this study, the MIDI function connected the pedalboard to an external sound module, enabling it to produce sounds through speakers that mimicked a traditional pipe organ. This feature was crucial in facilitating a realistic musical experience during the rehabilitation exercises.” |
| 9. Was member checking conducted? Member checking, also referred to as participant validation or respondent validation, is a technique employed in qualitative research to bolster the credibility and accuracy of the findings. During this procedure, researchers share their interpretations, analyses, or summaries of the data with the participants who contributed the data. The objective is to confirm that the researchers' depictions accurately reflect the participants' experiences, viewpoints, and interpretations. If member checking was performed, please describe the process. | Member checking was not performed and we have mentioned that in **Data analysis (page 13, lines 274-275)**: “The transcripts were not returned to the participants for comments and/or correction.” and **(page 13, line 284)**: “The participants did not provide feedback on the findings.”. This decision was made to streamline the research process and due to time constraints.  However, we acknowledged that the lack of member checking may limit the ability to confirm that the interpretations accurately reflect the participants' experiences and viewpoints. We address this issue as limitations by including the following text in **Discussion (page 37, lines 827-829)**:  “Meanwhile, member checking was not conducted due to time constraints. This may limit the ability to confirm whether our interpretations and findings accurately reflect participants’ viewpoints.” |
| 10. Was peer checking performed? Peer checking, also known as peer debriefing or peer review, is a process used in qualitative research to enhance the rigor and credibility of the study. This technique involves researchers sharing their data, analysis, or interpretations with colleagues or peers who have expertise in the field or in qualitative research methods. The purpose is to gain additional perspectives and ensure that the research process and findings are accurate, reliable, and free from bias. If it was performed, how? | Peer checking, also known as peer debriefing, was employed in this study to enhance the rigor and credibility of the research findings. The following text is added under **Data analysis (pages 13-14, lines 286-293)**:  “Peer checking was employed in this study to enhance the rigor and credibility of the research findings. The researchers MK and WK, both with substantial experience in qualitative research, independently analyzed the data. They engaged in regular discussions throughout the analysis process to compare their interpretations, resolve discrepancies, and refine the coding framework. These peer checking sessions helped ensure that the findings were robust, consistent, and free from individual bias. Additionally, the co-researcher (FA) provided critical feedback on the themes and interpretations, contributing to the overall trustworthiness of the study.” |
| Comments on the Results |  |
| 1. What is the homogeneity of the age and year of practice distribution? Should the median (IQR) be reported instead of the mean (SD)? | The analysis of the participants' demographic data reveals some variability in age and years of practice. We agree to report this information using median (IQR) instead, as following **(pages 15, lines 328-332)**:  “The median age of participants was 32.5 years, with an interquartile range (IQR) of 21 (50-29) years, indicating a relatively broad age distribution. Similarly, the median years of professional practice was 5 years, with an IQR of 6.5 (9.5-3) years. This distribution suggests a diverse range of experience levels among participants, with both early-career and more experienced professionals represented.” |
| 2. Please revise this that it clearly adds up to 17 participants. At the point in the text that reads “Each group consisted of one MT, one OT, one PT, and 1 one ST, except for one group, which included two OTs.” | We include the following text to improve clarity **(page 15, lines 322-326)**:  “The focus groups were composed as follows: three groups included one music therapist, one occupational therapist, one physiotherapist, and one sports trainer. One group included two occupational therapists, in addition to the other three disciplines, resulting in a total of 17 participants. This composition ensured that a variety of perspectives from different healthcare disciplines were represented.” |
| 3. Did the investigators obtain information regarding the participants' areas of expertise? | We did not obtain information on the participants’ area of expertise but include the major population of patients they cared for, as in Table 1 and in the text **(pages 15-16, lines 337-338)**:  “The participants primarily served older adult (n = 10), adult (n = 5), and paediatric (n = 2) patient populations.” |
| 4. What type of rehabilitation tool is this? Does it address emotional needs? At the point in the text that reads “Utilizing the music/organ pedalboard as a rehabilitation tool.” | The pedalboard is a musical instrument typically associated with organs and keyboards, consisting of a set of pedals that are played with the feet. In the context of our study, we are exploring the use of this pedalboard as a novel rehabilitation tool for lower extremity conditions, such as knee osteoarthritis. The key benefits of utilizing the pedalboard as a rehabilitation tool may go beyond just the physical aspects. By engaging the lower extremities in the rhythmic and coordinated movements required to play the pedalboard, it may address not only the physical impairments but also the emotional and psychological needs of the patients. The act of playing the pedalboard can provide a sense of accomplishment, enjoyment, and distraction from the pain and disability associated with conditions like knee osteoarthritis. The integration of music and the tactile experience of the pedals can have a positive impact on the patient's mood, motivation, and overall well-being.  Additionally, the pedalboard can serve as a platform for social interaction and engagement, particularly when used in a group rehabilitation setting. Patients may experience increased social connectedness and a sense of community, which can further enhance the emotional and psychosocial benefits of this rehabilitation approach.  However, to prove the effectiveness of the pedalboard as a rehabilitation tool, we need to conduct a study that explores not only the physical outcomes but also the psychological and social aspects. This would involve incorporating appropriate outcome measures that capture the multidimensional impact of this intervention on the patients' overall well-being and rehabilitation experience. |
| Comments on the Discussion |  |
| 1. It would have been interesting to determine if professionals' perspectives regarding the use of organ pedalboards align with the proposed framework, which is not presented in the Introduction. Understanding professionals' views on the use of organ pedalboards in relation to the proposed framework is essential for several reasons. Firstly, if professionals agree with the framework, it affirms the framework's relevance and effectiveness in practical settings. Their insights can reveal practical challenges, benefits, and nuances that may not be immediately evident from a purely theoretical standpoint. By incorporating these practical experiences, the framework can be refined to better address real-world applications. Moreover, professionals' feedback can lead to meaningful improvements and innovations, ensuring the framework remains relevant and effective. This alignment not only enhances the credibility of the framework but also increases the likelihood of its adoption and effective use within the field. In conclusion, incorporating the perspectives of regular users of organ pedalboards guarantees that theoretical frameworks are both academically rigorous and practically valuable. | We insert the following text in **Discussion** to interpret our findings with the Biopsychosocial Model **(pages 34-35, lines 751-771)**:  “This study highlights the importance of aligning healthcare professionals' perspectives with the theoretical framework proposed for the use of organ pedalboards in rehabilitation. The findings reveal that professionals' insights generally support its key components, particularly in relation to the Biopsychosocial Model. This model emphasizes the interplay of biological, psychological, and social factors in health, which are also reflected in the perceived benefits of organ pedalboard training.  The professionals agreed that the organ pedalboard could effectively address the biological aspects of rehabilitation by improving range of motion, flexibility, and coordination. These practical insights align with the framework's biological dimension, affirming its relevance in real-world applications. Additionally, the psychological and social benefits of using the organ pedalboard, such as enhanced patient motivation, cognitive engagement, and social participation, also resonate with the framework’s focus on these areas.  However, some discrepancies between theoretical expectations and practical challenges were identified. For example, while the framework posits that music-based rehabilitation tools could universally enhance emotional well-being, the professionals noted that patient preferences for music vary significantly, which could impact the tool's effectiveness. This feedback suggests a need for further refinement of the framework to account for individual differences in patient responses to music.  Incorporating these practical experiences into the framework would enhance its applicability and credibility, ensuring it addresses real-world challenges while maintaining theoretical rigor. Aligning the framework with the insights of regular users increases the likelihood of its successful adoption and effective use in rehabilitation settings, ultimately leading to better patient outcomes.” |
| Suggestion on Reflexivity |  |
| 1. It would be beneficial to include a section on reflexivity. Reflexivity in qualitative research involves the process of reflecting on and critically examining how researchers’ own backgrounds, perspectives, and interactions with the research process influence their study. This entails recognizing and disclosing potential biases, assumptions, and personal experiences that could shape the research design, data collection, analysis, and interpretation. Reflexivity is crucial because it provides transparency about the researcher's role and potential biases, thereby enhancing the credibility and trustworthiness of the research. By acknowledging their own biases and preconceptions, researchers can better account for how these factors might impact their findings and interpretations, leading to more balanced and objective analysis. Additionally, reflexivity promotes ethical considerations by addressing power dynamics and the researcher-participant relationship, encouraging a more respectful and mindful approach to research. Reflexivity is typically addressed in the Methodology section of a manuscript, where researchers discuss their background and how it might influence the research process. However, it can also be mentioned in the Introduction to provide context or revisited in the Discussion section to reflect on how the researcher’s perspectives impacted the findings. Overall, reflexivity is a critical aspect of qualitative research as it ensures that the study is conducted with awareness and integrity, contributing to more credible and insightful results. | Reflexivity in this study was a critical aspect that helped ensure the credibility and trustworthiness of the findings. We add a section of **Reflexivity** in **Methods** to address the issues surrounding personal reflexivity and relational reflexivity **(pages 14-15, lines 296-318)**:  “The research team, led by MK, who has a background as a registered nurse with a Master’s degree in psychology of music, was deeply aware of how personal experiences and professional background could influence the research process. The team consciously reflected on their assumptions about the use of music and organ pedalboards in rehabilitation. Recognizing that their professional backgrounds could predispose them to view these interventions positively, the researchers employed strategies such as peer checking and collaborative data analysis to challenge their interpretations and ensure a more balanced analysis. This reflexive approach contributed to a deeper understanding of how the researchers' perspectives might have shaped the study's design, data collection, and analysis. By acknowledging these influences, the research team enhanced the study's transparency, thereby increasing the credibility and reliability of the findings.  Reflexivity was integral to the ethical conduct of this research, as it helped minimize power dynamics and ensure the participants' voices were accurately represented, leading to balanced and objective conclusions. Given that some participants were personally acquainted with the lead researcher (MK), particular measures were employed to minimize potential bias. All participants were explicitly informed of the study's objectives and the distinct role of MK as a facilitator, separate from any personal connection. Participants were encouraged to provide honest and candid feedback, independent of their relationship with MK. Additionally, transparency was reinforced by emphasizing the structured nature of the interview process, which strictly adhered to the interview guide developed by the research team. During the interviews, MK maintained a neutral stance and carefully managed her influence to minimize bias.” |
| I pray the comments help in improving the manuscript. I look forward to reading the updated manuscript. Stay Blessed, | Thanks you for your praying and wishes. |
