# Supplementary material for: Organ pedalboard as a rehabilitation tool: A qualitative exploratory study of healthcare providers’ perceptions and recommendations
Source: PLoS One. 2024 Dec 19;19(12):e0314147. doi: 10.1371/journal.pone.0314147 (PMC11658632; doi:10.1371/journal.pone.0314147)
Supplement: Attachment — Submitted filename: Response to Reviewers.docx [file pone.0314147.s004.docx]

**Responses to Reviewer #1’s comments:**

| **Comments** | **Responses** |
| --- | --- |
| I would like to thank the authors for their efforts in the revision. The manuscript has been improved significantly in terms of conceptual base, methodological credibility, clarity of findings, and critical discussion. Here are a few further points for authors’ consideration: | Thank you very much. Please refer to our responses below. |
| 1. The identification of higher-order themes helped to present the findings clearly. I may suggest that the higher-order themes can be more concise. For example, the second higher-order theme can be “Benefits of Organ Pedalboard”, and the third one can be “Modifications of Organ Pedalboard”. Also, the wordings in the two sub-themes under the first higher-order theme appear to be similar. It is not easy to differentiate these two without reading the text and quotes. | Thank you for your suggestion.  We changed the second higher-order theme to “Benefits of Organ Pedalboard” (lines 354, 421) and the third higher-order theme to “Modifications of Organ Pedalboard” (lines 361, 544).  We also changed the two sub-theme wordings of the first higher-order theme to “Music facilitates rehabilitation” (line 370) and “Detaching patient role through music rehabilitation” (lines 399-400), respectively. |
| 2. The discussion on the issue of cost (Lines 773-784) is critical and valid. However, it seems more important to discuss why this theme was not extensively discussed during the focus group interviews and how future studies may address this. The authors mentioned that it was probably due to “participants’ primary focus…on the therapeutic advantages”. It is important to interpret why they had such primary focus. Possible influential factors may include the project team’s underlying assumptions of therapeutic effects of organ pedalboard, limited time/session for focus group interviews, or the role of the participants (as health professionals rather than managers of health services). It is a good suggestion of applying organ pedalboard through institutional ownership, but may consider soften the tone, making this a possible direction for future study. | Thank you for your comment. We added the followings to explain further:  “The participants' primary focus was on the therapeutic advantages rather than the financial barriers. It was because most of the participants worked under an organization. They used tools provided by the organization to manage their patients. Understanding the therapeutic effects of pedalboard for patients enabled participants to recommend this tool to the decision makers of their workplace.” (lines 774-777)  Also, we refined the sentence about the suggestion of institutional ownership:  “Future research could investigate the feasibility of this institutional ownership approach. This strategy is anticipated to enhance accessibility and alleviate individual financial burdens, allowing the primary focus to remain on the clinical benefits of this rehabilitation tool.” (lines 782-785) |
| 3. Like the issue of cost, the authors may also interpret why cultural barriers (Lines 786-805) was not covered in the findings of the study. Also, may consider soften the tone about the point of “train-the-trainer”, making it a possible future direction of research. | Since numerous topics were covered in this exploratory study, we did not have rooms to in-depth evaluate the cultural factors regarding the pedalboard adoption during the focus group interviews. We added a sentence to explain this: “However, we did not discuss any cultural factors influencing its adoption during this exploratory study due to time constraints.” (lines 787-788).  Meanwhile, we acknowledged that cultural factors could influence the pedalboard adoption. We suggested that future research could explore such factors by understanding patients’ perceptions and attitudes directly, rather than from the health professionals. For example, in lines 804-808: “Importantly, future research should also explore patients' perceptions, attitudes, and experiences with the organ pedalboard directly. Understanding the target population's perspectives, preferences, and cultural considerations will be crucial in developing effective strategies for the successful integration of this rehabilitation tool.”  We also refined the sentence about the point “train -the-trainer”:  “We recommend that future research assess the potential effectiveness of integrating the organ pedalboard into rehabilitation settings under institutional ownership, along with the implementation of 'train-the-trainer' programs for healthcare professionals.” (lines 800-802) |
| 4. In the discussion of generalizability (Lines 807-815), the authors mentioned that “We believe that there are minimal barriers to pedalboard rehabilitation adoption in similar developed regions globally”, and this seems stretching too far. The findings of the study can be applicable to the regions with sociocultural context similar to Hong Kong. But it does not mean that organ pedalboard can be applied successfully, with minimal barriers. | We agreed that the original sentence was illogical. We added the following sentence (lines 811-8112):  “We believe that the findings of the study can also be applicable to the regions with sociocultural context similar to Hong Kong.” |
| 5. Here may be some typos:  Lines 131-139: “The repetitive goal-oriented nature to improve functional outcomes through tasks that mimic daily activities” Do you mean “The repetitive goal-oriented nature can improve functional outcomes through tasks that mimic daily activities” ?  Lines 807-815: “The findings offer valuable insights, but they should be interpreted within Hong Kong's sociocultural.” Do you mean “…but they should be interpreted within Hong Kong's sociocultural context” ? | Yes, thanks for spotting these errors. We have made corrections to both sentences (line 136 and line 811). |

**Responses to Reviewer #2’s comments:**

| **Comments** | **Responses** |
| --- | --- |
| Thank you for thoroughly addressing the provided comments and suggestions. I recommend that your revised manuscript be accepted for publication in PLOS ONE. Congratulations! | Thank you very much and we wish you the best. |
